# Preclinical studies of non-stick thin film metallic glass-coated syringe needles

**DOI:** 10.1038/s41598-020-77008-y

**Published:** 2020-11-20

**Authors:** Meng-Yi Bai, Ya-Chu Chang, Jinn P. Chu

**Affiliations:** 1 Graduate Institute of Biomedical Engineering, National Taiwan University of Science and Technology, No. 43, Keelung Rd., Sec.4, Da’an Dist., Taipei City, 10607 Taiwan; 2 Adjunct Appointment to the National Defense Medical Center, Taipei City, 11490 Taiwan; 3 Department of Materials Science and Engineering, National Taiwan University of Science and Technology, No. 43, Keelung Rd., Sec.4, Da’an Dist., Taipei City, 10607 Taiwan; 4 Applied Research Center for Thin-Film Metallic Glass, National Taiwan University of Science and Technology, Taipei City, 10607 Taiwan

**Keywords:** Materials science, Nanoscience and technology

## Abstract

Our objective in this study was to determine the biocompatibility and hemocompatibility of thin film metallic glass (TFMG) and its potential use in hypodermic needles for intramuscular or intravenous injection. Mouse and rabbit models were employed under approval from the Institutional Animal Care and Use Committee (n = 5/group, two groups in total for both animal models). Platelet-rich plasma (PRP) was collected from the whole blood of rabbits (ear vein) without anti-coagulant for use in in vitro coagulation tests. Histological analysis and optical microscopy were used to assess the endothelial structure of the inner lining of veins after being punctured with needles and detained for 3 days. Histological analysis of ear vein sections revealed that the extent of endothelial damage after puncturing with a TFMG-coated needle was 33% less than that produced by bare needles. Our results confirm that the deposition of a thin TFMG layer (e.g., Zr_53_Cu_33_Al_9_Ta_5_) on the surface of hypodermic needle can have remarkably clinical benefits, including anti-adhesion, reduced invasion, and minimal endothelial damage. Our results also confirm the good biocompatibility and hemocompatibility of the TFMG coatings.

## Introduction

Disposable hypodermic needles are among the medical devices most frequently used for intravenous or intramuscular injection^1^. They can be used to obtain liquid samples from the body or deliver drugs into the body. Disposable hypodermic needles were introduced in the early 1950′s for the administration of morphine and penicillin, and they have increased in popularly, particularly in response to the HIV epidemic since the 1980s^1^. Hypodermic syringes are the first point of contact with a biological system when conducting injection and must therefore be made of biocompatible materials that are pharmacologically inert, sterilizable, and nontoxic^2^. Stainless steel (e.g., ASTM 45500) is the material most frequently used for medical devices, due to its excellent biocompatibility and inertness; however, stainless steel is not defect-free. Any close inspection of stainless steel surfaces is bound to reveal a large number of scratches, which tend to promote cell adhesion. In previous studies, we demonstrated the benefits of applying thin film metallic glass (TFMG) to surgical blades and needles in terms of antibacterial properties, sharpness, and durability^3–6^. Metallic glasses (MGs) (i.e., amorphous metallic alloys) are noncrystalline metals, which lack long-range atomic periodicity associated with the rapid quench rates used in fabrication. The first reports of metallic glass were presented by Klement et al.^7^, Chen and Turnbull^8^, and Chen^9^ in the 1960s and 1970s. In recent decades, the application of TFMGs has focused primarily on antimicrobial performance. There has been a great deal of work on Zr-based TFMGs containing Cu [e.g., Zr61Cul7.5Ni10Al7.5Si4)] and Ag [e.g., (Zr42Cu42Al8Ag8)99.5Si0.5]^10–12^. Nonetheless, these materials do not really provide any benefits beyond their antimicrobial properties. Researchers have demonstrated the application of Zr-based TFMGs to medical tools, such as surgical blades and micro-surgery scissors. Most commercial blades are made of martensitic stainless steel, which is prone to micron-scale imperfections on the surface and along the edge^13^. This kind of roughness can greatly reduce blade sharpness, exacerbate cell adhesion, undermine incision quality, and reduce the lifespan of the tool. However, we discovered a new combination of TFMG (Zr_53_Cu_33_Al_9_Ta_5_), with very low surface roughness. (after coating: with ~ 10.0 ± 1.7 nm) when coating on needle (before coating: with ~ 16.2 ± 2.7 nm average roughness). The proposed TFMG has high strength, superior toughness, and excellent hydrophobicity associated with low surface free energy. Applying a thin layer of TMFG to the surface of 31 G SS304 needles was shown to greatly reduce adhesion, increase hydrophobicity, and decrease friction to levels far below those of bare stainless steel needle. TFMG-coated needles are intended for intravenous and/or intramuscular injection in living systems; therefore, previous investigations based on phantom or porcine skin models were insufficient to obtain a reasonable assessment of their applicability. TMFG-coated needles have been extensively evaluated in terms of anti-microbial performance; however, the applicability, safety, and biocompatibility of these coatings have not been assessed in vivo^14–16^. In previous in vitro analysis, we demonstrated the effectiveness of TFMG-coated needles in piercing porcine skin samples. Herein, in vivo musculoskeletal animal model further demonstrated the potential of TFMG-coated needles in intravenous or intramuscular injection. Previous mouse and rabbit models^17^ were respectively used to demonstrate the efficacy of various biomaterials in wound management and harvesting platelet cytokines.

In this study, we launched a pilot preclinical trial to ascertain whether TFMG-related materials (in the form of TFMG-coated needles), could outperform bare stainless steel when used for intravenous and intramuscular injection. We then harvested previously used TFMG-coated needles and full skin samples surrounding the injection site for histological staining to visualize tissue debris associated with adhesion and the puncture wounds created by piercing. Our results obtained from a small animal model were then compared with previous findings based on phantom and porcine skin models^18^. We also inserted a TFMG-coated scalp vein needle into the ear vein of New Zealand rabbits to assess the hemocompatibility of the TFMG material and its effects on endothelial cells in blood vessels. Although Mg-based TFMG is known for its good biodegradable property, the Mg-based TFMG is known having low Tg (glass-transition temperature) and weak in strength. Hence, it is not suitable to be used in the present study as the coating on the needle. During insertion of needle back and forth into the skin, the robust and strong coating is thought an important criterion, to avoid any worn-out and possible peel-off.

Our analytical results clearly demonstrated the beneficial effects of TFMG-coated needles in terms of adhesion, durability, invasiveness, and hemocompatibility. This material has very strong potential for use in clinical applications, such as needles for injection. To the best of our knowledge, this is the first work reporting the functional performance of TMFG-coated medical devices using a small animal model.

## Results

### Composition and surface properties of TFMG-coated hypodermic needles

**Figure 1:**
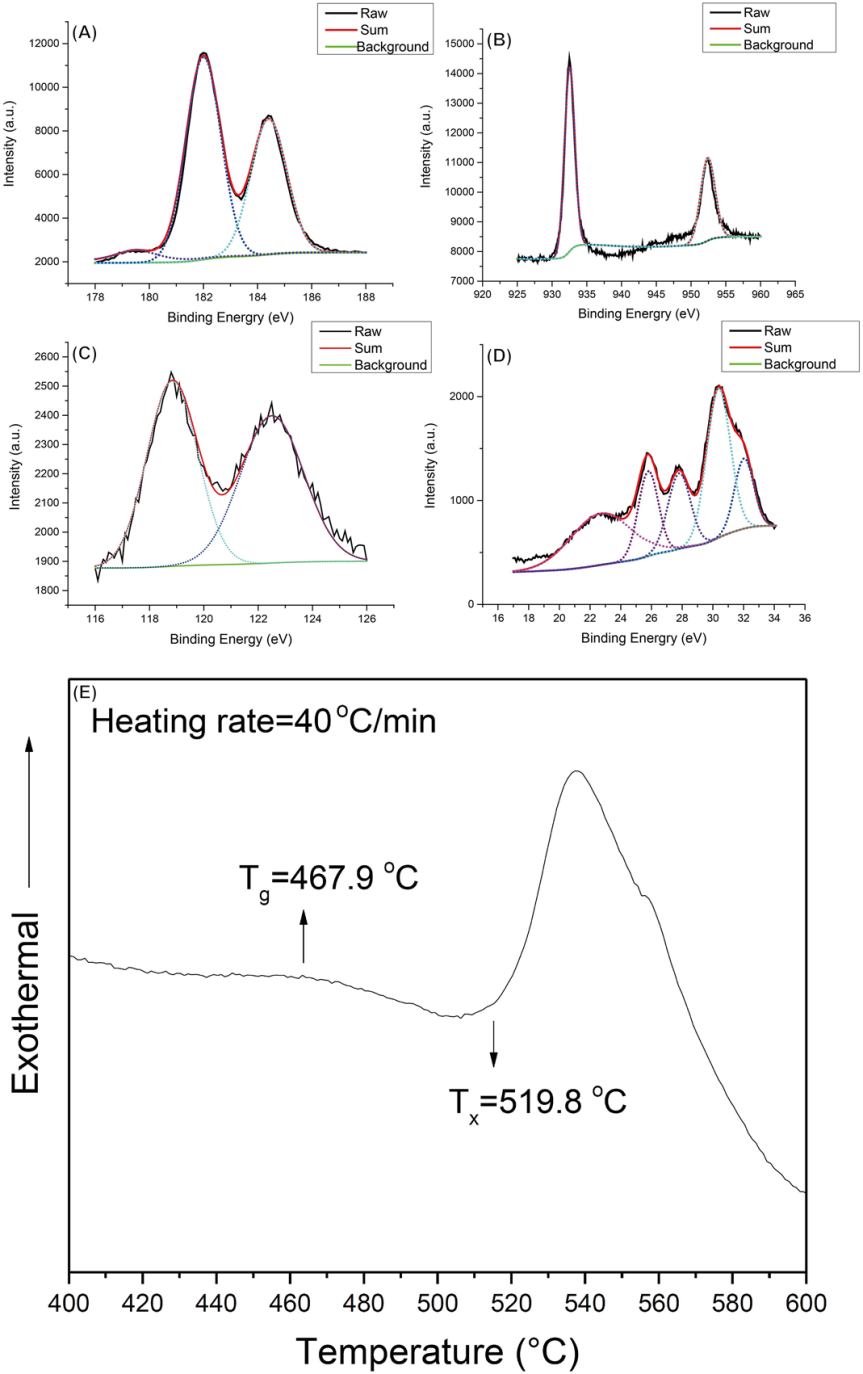
Results of (**A**–**D**) high-resolution X-ray photoelectron spectroscopy (XPS) and (**E**) differential scanning calorimetery (DSC). The four main constituents of the TFMG coating layer were as follows (in atomic percentage): Zr (53.0%)–Cu (32.8%)–Al (9.0%)–Ta (5.2%). The structure of the coating material was confirmed as amorphous.

The surface composition and crystallographic properties of the TFMG-coated hypodermic needles were respectively characterized using high-resolution X-ray photoelectron spectroscopy (XPS) and differential scanning calorimetry (DSC) (Fig. 1). The four main constituents of the TFMG coating layer were as follows (in atomic percentage): Zr (53.0%)–Cu (32.8%)–Al (9.0%)–Ta (5.2%). These values are very similar to those of the metal target plate used for sputtering. X-ray diffraction (XRD) was used to characterize the crystalline state of the TFMG coating layer. The results revealed a broad peak ranging from approximately 30° to 50° without detectable crystalline diffraction peaks. This is a clear indication that the structure of the film was amorphous. The DSC results in Fig. 1E reveals a typical glass transition (T_g_) at 467.9 °C and exothermic event of crystallization (T_x_) at 519.8 °C. These observations provide solid evidence that the TFMG coating layer on the hypodermic needles was an amorphous form of Zr _53_Cu_33_Al_9_Ta_5_.

### In vitro cytotoxicity assays of TFMG-coated hypodermic needles using 3T3 fibroblast cells

**Figure 2:**
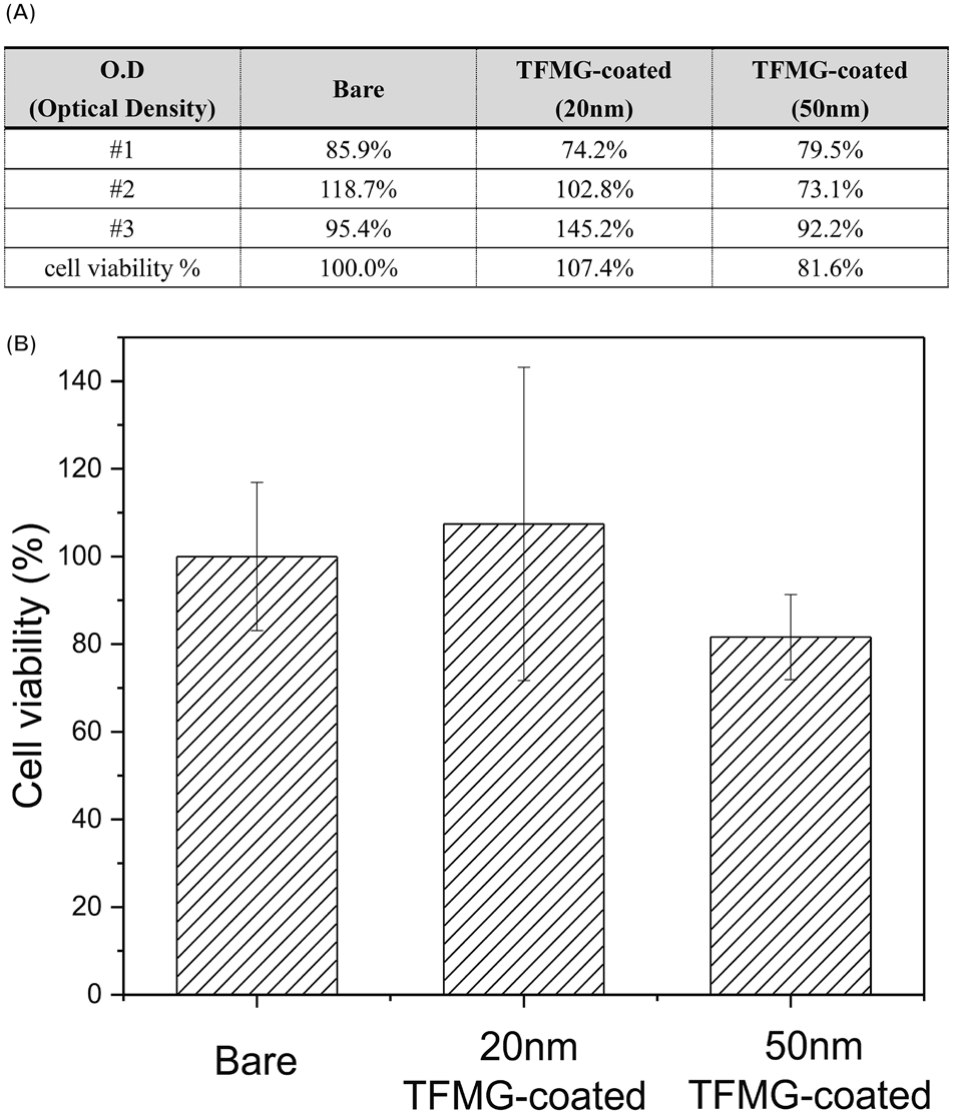
MTT assays to test the cytotoxicity of bare stainless steel samples and a TFMG-coated samples (thickness of 20 nm or 50 nm) toward 3T3 fibroblast cells: (**A**) optical density of all individual results and (**B**) after normalization to the value of bare stainless steel needles (control group).

**Figure 3:**
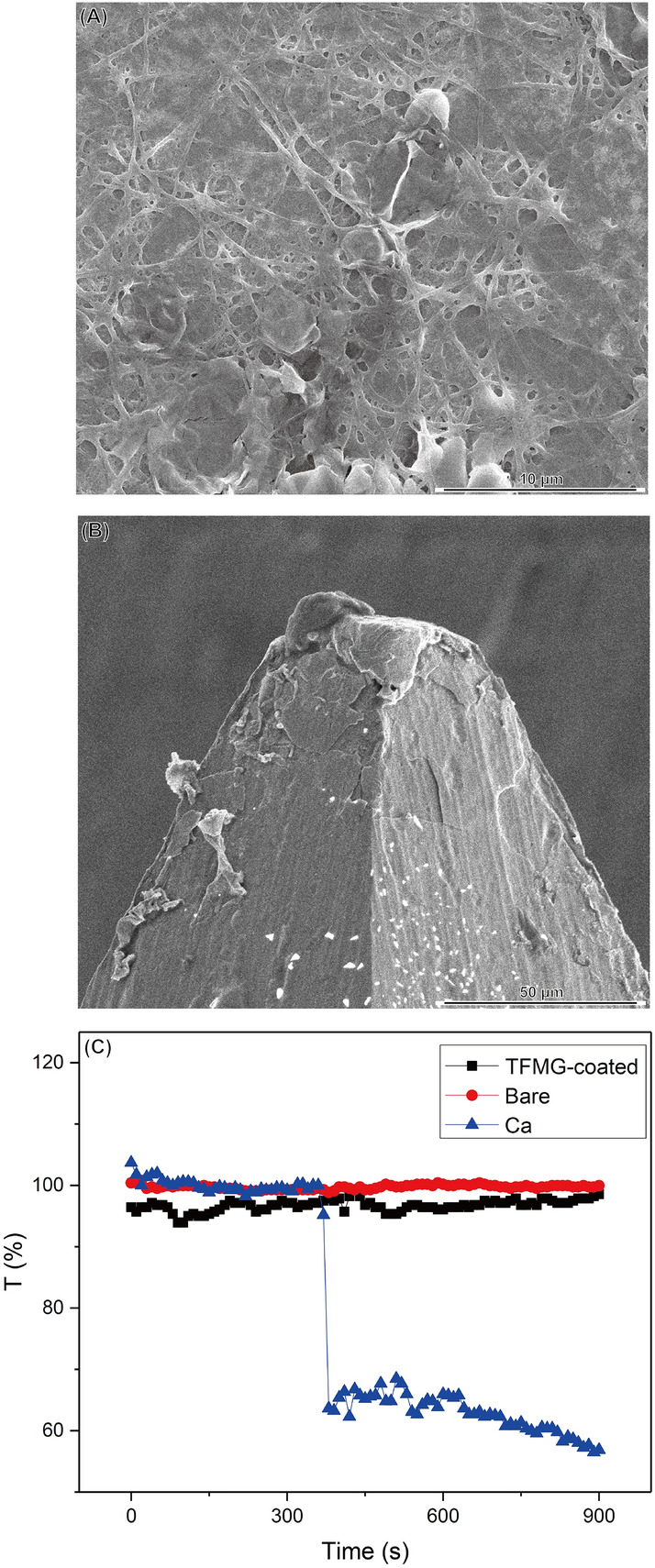
Test of thrombosis using platelet-rich plasma freshly harvested from New Zealand rabbits: (**A**) SEM image of PRP coagulation clots obtained by inducing coagulation in PRP, (**B**) surface of the TFMG-coated and (**C**) coagulation test of bare, TFMG-coated hypodermic needles, and positive control group (with the addition of calcium ions).

Hypodermic needles are used for the delivery of pharmaceutical substances into biological systems; therefore, contact with body fluids is inevitable, as is the leaching of potentially toxic molecules or ions. In the current study, we employed elution assays to model the biological environment. Briefly, this approach involved the elution of soluble molecules from a test item into an aqueous media. Figure 2A,B present a series of MTT assays using 3T3 fibroblast cell to evaluate the cytotoxicity of bare needles and TFMG-coated hypodermic needles (coatings of 20 or 50 nm). The viability of cells exposed to TFMG-coated needles was 100 ± 20% (20 nm coating) and 81.6 ± 10% (50 nm coating), compared to that of bare hypodermic needles. Based on ISO 10993-5 standards, cell viability exceeding 80% is considered non-cytotoxic. This is a clear indication that the TFMG-coated hypodermic needles are safe to pierce the skin and muscle for intramuscular injection. Hemocompatibility is another issue of critical importance in the evaluation of hypodermic needles for intravenous injection. We adopted the ISO 10993-5 standards in our selection of biocompatibility tests for medical devices. Figure 3A presents the test used to evaluate thrombosis in platelet-rich plasma freshly harvested from New Zealand rabbits. An optical microscope was used to visualize the formation of adhered platelets, leukocytes, aggregates, erythrocytes, generation of fibrin and a scanning electron microscope (SEM) was used to characterize platelet morphology following the immersion of needles over a period of 15 min. As shown in Fig. 3B, very few platelets were adhered to the TFMG-coated needles and there was no evidence of fibrin formation (see Fig. 3A for comparison). Figure 3A, B present qualitative test results and Fig. 3C presents quantitative analysis based on the transmittance of visible light. The curves obtained from bare and TFMG-coated samples both reached nearly 100% of transmittance within 15 min of detection time; however, the initial reduction in transmittance in the control group was far more rapid. (calcium ions were added, a role of inducing coagulation in intrinsic and extrinsic coagulation pathway), This clearly demonstrates that the hemocompatibility of the TFMG-coated needles was comparable to that of the bare needles. Overall, these findings indicate that the TFMG-coating did not induce platelet aggregation, activation and fibrinogen polymerization, which could potentially be lethal.

### In vivo test of cell and tissue adhesion using a mouse model

**Figure 4:**
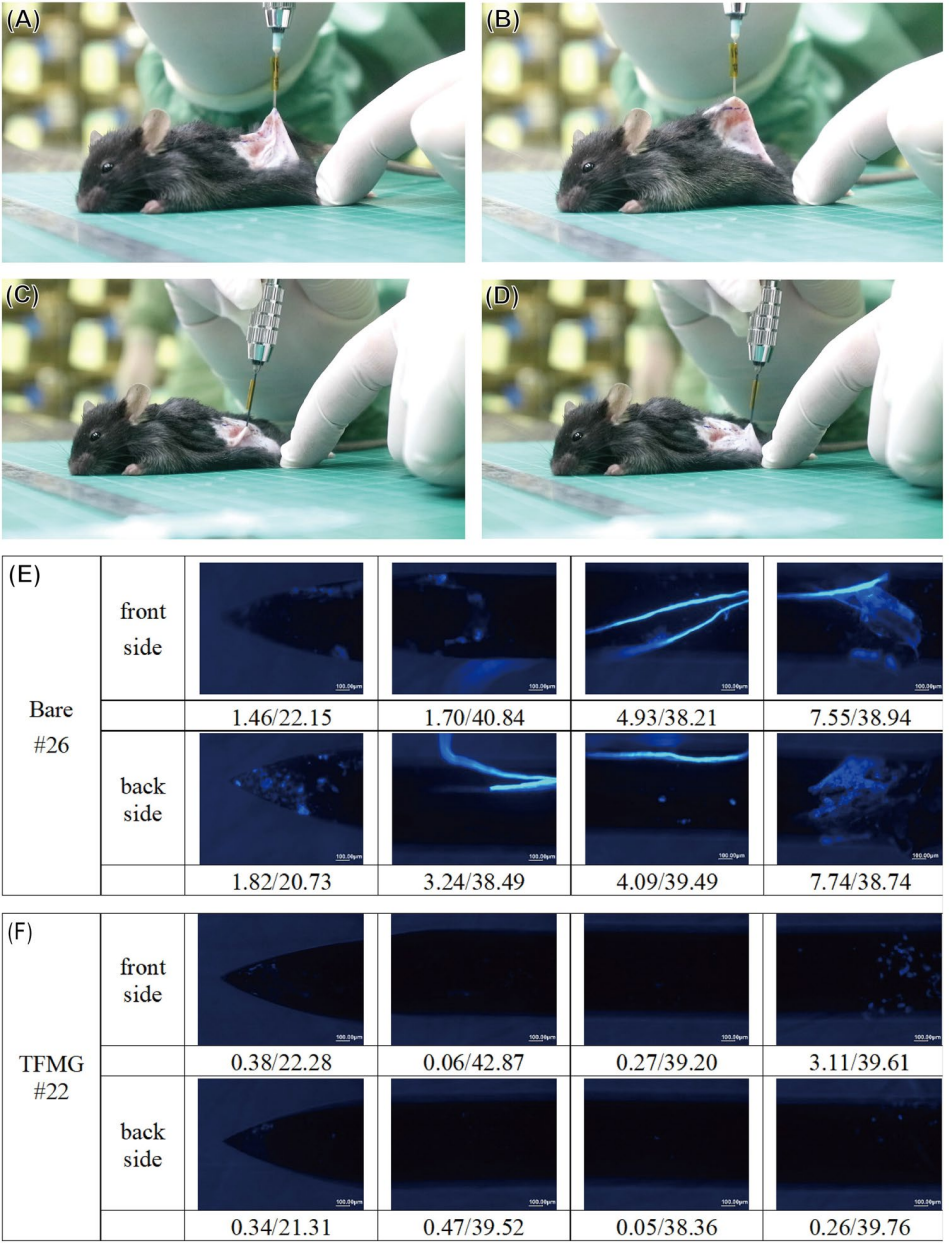
(**A**–**D**) Optical photographs obtained during the puncturing of mouse dorsal skin: (**A**,**B**) bare stainless steel hypodermic needles; (**C**,**D**) TFMG-coated hypodermic needle group; (**E**,**F**) representative whole images of used bare and TFMG-coated hypodermic needles after being subject to DAPI staining, respectively.

**Figure 5:**
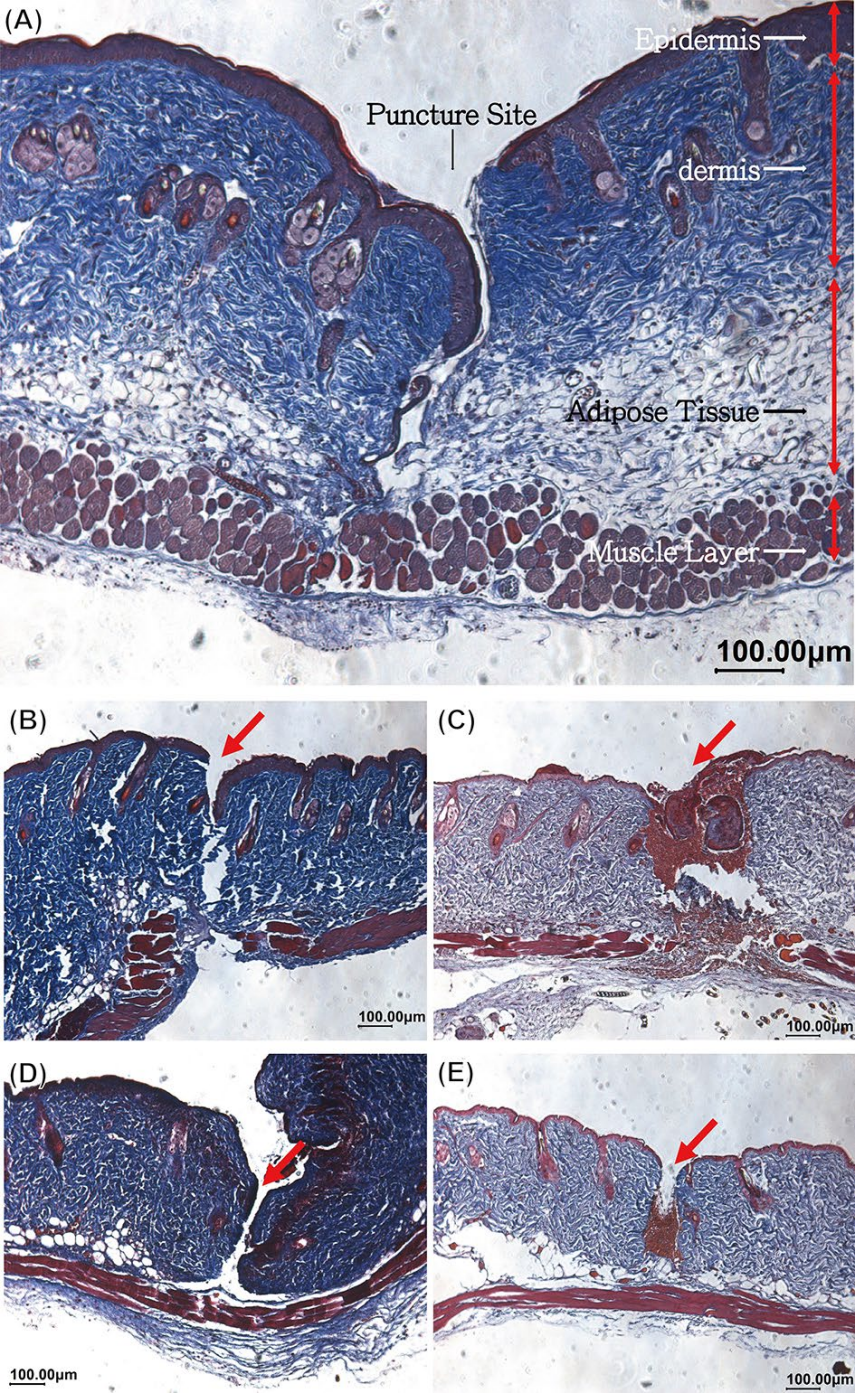
Representative Masson staining images of mouse skin after being punctured by (**A**,**D**,**E**) TFMG-coated hypodermic needles and (**B**,**C**) bare stainless steel hypodermic needles.

In a previous study, we clearly demonstrated the beneficial effects of thin film metallic glass coatings on syringe needles in terms of their non-stick behavior in pork muscle tissue (reduced insertion and retraction forces when using TFMG coatings). In this study, we extended our analysis of cell-adhesion and non-stick performance using an in vivo mouse model based on the physiological feature2 that mouse skin is very similar to that of human skin. Figure 4A–D presents photographs showing the puncturing of mouse dorsal skin by bare and TFMG-coated hypodermic needles. As shown in Fig. 4A, B, tissue clearly stuck to the bare needles during retraction. Note that the area of elevated tissue in the image is referred to as a hump. The lack of an observable hump in the image of TFMG-coated needle is a clear illustration of non-stick characteristics of TFMG (Fig. 4C, D). All of the used needles (bare and TFMG-coated) were collected after they had each been used for puncturing 20 times. They were then stained with 4′,6-diamidino-2-phenylindole (DAPI) reagent to pinpoint cells that had adhered to the surface of the needles. Figure 4E, F present representative images of the needles after being subjected to DAPI staining. Table 1 summarizes the statistic results of cell adhesion as derived from the area of fluorescence on the DAPI stained needles. Cell adhesion in the TFMG-coated group was 79.6% lower than that in the bare needle group (n = 3 mouse). Please refer to Figure S2 for the raw statistical data. This remarkable resistance to cell adhesion can be attributed to the non-stick behavior shown in Fig. 4C, D. To the best of our knowledge, the plausible mechanism of anti-adhesion performance of the TFMG is originated from the smooth and grain boundary-free surface, as well as the low surface energy. Our recent publication has been shown that the Zr-based TFMG selected in the present study has the lowest surface energy among many other TFMG systems (26.90 mN/m)^19^. Dorsal skin from the mice was also harvested after scarification for histological analysis. Figure 5A presents representative Masson staining images of the skin after being punctured by TFMG-coated hypodermic needles. We can see that the puncture extended through the top epidermis layer into the bottom muscle layer. A close examination of the wall of the puncture channel created by the bare hypodermic needles revealed a deckle edge appearance (as indicated by the red arrowhead in Fig. 5B, C). In contrast, the puncture channel created by the TFMG-coated hypodermic needles presented smooth, intact edges (as indicated the red arrowhead in Fig. 5D, E). Based on the results in Fig. 4, we believe that the ease of retraction is responsible for the uniformity of the puncture wounds in Fig. 5. We also derived quantitative (statistical) results of wound size, as estimated from Masson staining images of mouse skin after biopsy. The average wound size (n = 8) was as follows: bare hypodermic needles (1.35 ± 1.92 mm^2^) and TFMG-coated hypodermic needles (0.72 ± 0.48 mm^2^) (see Figure S3 for the raw data). This indicates that the TFMG-coated hypodermic needles enabled a 46.7% reduction in wound size compared to the bare needles.

**Table 1:** Statistic results of adhesion cell assessed from the fluorescence area on the DAPI stained needles after being subject to 20 repeats of puncture.

Material	Bare (stainless steel)			TFMG		
Mice label	#1	#2	#3	#1	#2	#3
Front side (%)	10.76	1.58	3.99	2.60	0.89	2.64
Back side (%)	11.90	3.84	5.93	0.89	0.43	0.29
Avg. of above (%)	11.33	2.71	4.96	1.74	0.66	1.46
Avg. of #1–3 ± SD	6.33% ± 4.47%			1.29% ± 0.56%

### In vivo hemocompatibility test of TFMG-coated hypodermic needles using rabbit model

**Figure 6:**
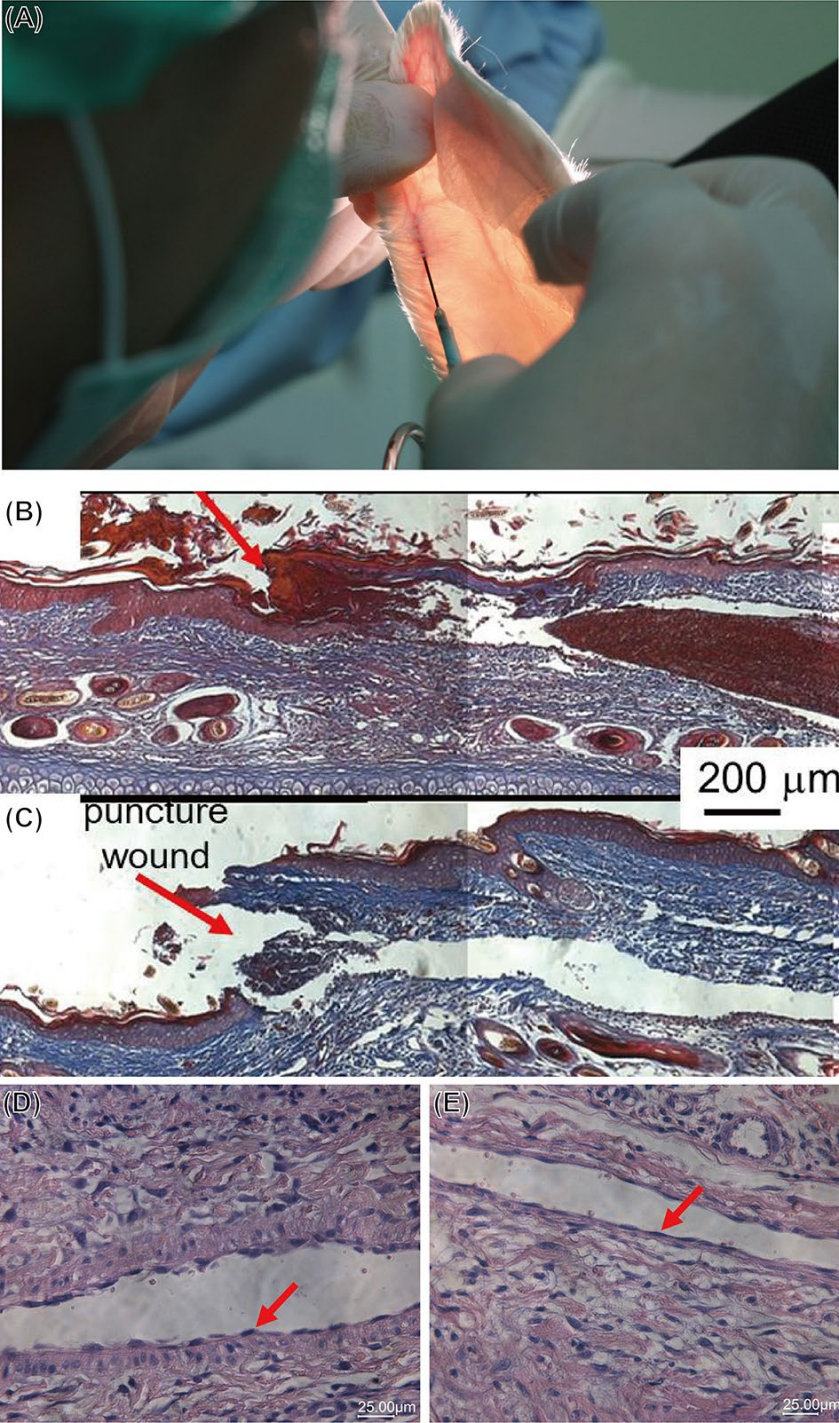
(**A**) Optical photograph indicating the position of the ear vein and method used to detain needle; (**B**,**C**) histopathological biopsies of puncture wound in ear vein of rabbit after bare or TFMG-coated needle was detained for 3 days, respectively. (**D**,**E**) enlarged images of the inner wall of the vein vessel from (**B**,**C**). Two different types of endothelial cells were observed.

The previous in vitro study on hemocompatibility using rabbit platelets ruled out the possibility of thrombogenicity; however, needles are often left in the vein for an extended period during the administration of infusions. Thus, we assessed the hemocompatibility of TFMG-coated hypodermic needles toward endothelial cells by examining the induction of vasculitis. Bare and TFMG-coated hypodermic needles were detained in the ear vein of rabbits for a period of 3 days, which is typical of the duration recommended for the replacement of vein detained needles. Figure 6A indicates the position of the ear vein used for the insertion of TFMG-coated hypodermic needles. Figures 6B, C respectively present the histopathological biopsies of puncture wounds from bare and TFMG-coated needles in the ear vein of rabbits after being detained for 3 days. In this analysis, we observed a pinhole in the epidermis layer extending only down to the dermis layer; i.e., we did not observe the muscle-deep penetration observed in the previous in vivo cell adhesion tests as the vein vessel is the target in the intravenous injection or detainment. Many disk-like red blood cells filled the channel of the vein vessel. A close examination of the inner wall of the vein vessel (Fig. 6D, E) revealed two types of endothelial cells: (1) innate endothelial cells with a flat and well-attached morphology (red arrowhead in Fig. 6E) and (2) newly regenerated endothelial cells with quasi-round and rolling morphology (red arrowhead in Fig. 6D). One plausible mechanism for the appearance of newly regenerated endothelial cells is damage to the inner lining of the vein walls caused by the rough surface of the needle during insertion. We therefore hypothesize that a needle with a smooth surface (e.g., a TFMG-coated hypodermic needle) should have less effect on the lining of the vein due to a reduction in the pull-and-drag effect. Our quantitative results clearly show that using TFMG-coated needles for vein detained injection resulted in fewer newly regenerated endothelial cells than did the bare needles. Figure 6D, E and Table 2 present the statistical results obtained from Masson stained biopsy images based on estimation of the length of innate and newly regenerated endothelial cells. Note that the average length of venous wall damage was as follows: bare needle group (32.97% ± 9.09) and TFMG-coated needle group (21.99% ± 7.56).

**Table 2:** Estimation of the cell length of innate and newly regenerated endothelial cells based on the Masson stained biopsies images.

Material	Bare				TFMG			
Vein damage length ratio (%)	27.76	25.01	30.32	34.21	32.46	26.52	25.72	21.18
	38.17	48.72	53.69	38.94	20.55	21.05	20.22	20.43
	26.44	34.04	33.31	37.03	28.45	35.22	20.12	12.33
	18.72	18.17	32.98	28.98	38.66	32.15	24.51	28.47
	22.94	36.37	37.17	31.89	13.14	8.70	8.62	12.29
	34.07	17.53	45.32	24.68	15.37	13.52	21.58	21.70
	36.92	43.91			20.78	22.57	23.03	30.30
					15.78	24.19		
Average length ratio (%)	32.97 ± 9.09				21.99 ± 7.56			

## Discussion

This study confirmed the efficacy of hypodermic needles coated via sputtering with a special class of TFMG coating (Zr_53_Cu_33_Al_9_Ta_5_) in terms of safety, intrusiveness, and biocompatibility. The main constituents of the TFMG coating layer were as follows (in atomic percentage): Zr (53.0%)–Cu (32.8%)–Al (9.0%)–Ta (5.2%) in an amorphous state. MTT cell assays demonstrated that the use of TFMG-coated hypodermic needles to extract medium resulted in cell viability of nearly 81–100%, indicating the absence of toxic ions or molecules leaching from the TFMG coating. PRP harvested via centrifugation at 700 rpm (60 g) for 3 min was used to evaluate the hemocompatibility of the TFMG-coated hypodermic needles in vitro. We observed no indications of thrombosis, and the morphology of the platelet was spherical (no pseudopodia was observed), thereby demonstrating the good hemocompatibility of the TFMG-coated hypodermic needles^20^. The transmission of visible light (414 nm) less than 5% reduction was detected, which indicates that no coagulation occurred to block the passage of light. In an in vivo study, we employed the detained needle method to test the hemocompatibility of TFMG material when in direct contact with endothelial cells*.* Previous researchers^21^ have clearly demonstrated that endothelial impairment is strongly tied to the occurrence of peripheral vascular disease, stroke, heart disease, diabetes, insulin resistance, chronic kidney failure, tumor growth, metastasis, venous thrombosis, and severe viral infection. Thus, it was essential that we obtain direct contact between the TFMG coating and the endothelium in the evaluation of TFMG-coated needles in terms of clinical safety. The proposed TFMG-coated needles presented outstanding anti-adhesion properties and minimal invasiveness, which make them ideally suited to the scalp veins used for the vein detained injection of infants.

In previous studies on the endothelium^21^, researchers determined that the creation of lesions on the endothelium lining leads immediately to the establishment of a functional vascular network. When stimulated by high levels of vascular endothelial growth factor (VEGF), endothelial cells derived from mesodermal precursors undergo morphological changes from round colonies to long elongated structures attached securely to the venous wall. In this study, we adopted this morphological difference between newly regenerated endothelial cells and intrinsically healthy endothelial cells as indicators by which to evaluate the extent of damage created during the insertion and/or retraction of needles. Compared to the control group, the TFMG-coated hypodermal needles reduced vein endothelial damage by 33%, thanks to the smooth surface of the TFMG coating. This also helped to minimize the size of the resulting puncture wound.

## Conclusion

In conclusion, this was the first systematic study to evaluate the biological safety of TFMG materials on medical devices. We found that the application of Zr_53_Cu_33_Al_9_Ta_5_ coatings to a thickness of 20–50 nm on the surface of medical devices (e.g., hypodermal needles) was sufficient to improve the durability and functional performance in terms of reduction in cell adhesion, invasiveness, and endothelial damage. In vitro and in vivo studies revealed that the Zr_53_Cu_33_Al_9_Ta_5_-based TFMG coating layer is not susceptible to the leaching of potentially toxic ions and is highly hemocompatible. We also determined that the uniformity and hydrophobicity of the TFMG coating greatly reduced the adhesion of biological tissue, compared to bare stainless steel samples. The proposed TFMG coating layer is ideally suited to nearly any type of medical device. We are currently investigating a wide variety of applications for the Zr_53_Cu_33_Al_9_Ta_5_-based TFMG coating. Furthermore, this coating technology would also be applicable to a wide range of consumer products, such as cutting implements^22^.

## Materials and methods

### Materials

23 G needles (scalp vein set, NIPRO, Japan) were used directly as the substrate for the deposition of a TMFG layer via magnetron sputtering. Thiazolyl blue tetrazolium bromide (MTT, 98%, Sigma-Aldrich, USA) and dimethyl sulfoxide (DMSO, reagent grade, Sigma-Aldrich, USA) were used in MTT assays. 1X PBS solution was prepared using phosphate buffer saline (PBS, 10×, Uni Biotech, Taiwan) by diluting 1 mL of 10× PBS in 9 mL of deionized water and stirring for 10–15 min.

### Preparation of TMFG-coated hypodermic needles

A Zr-based (Zr53Cu33Al9Ta5) target with a diameter of 3 inches was used to deposit TFMG on the substrate using radio frequency (RF) magnetron sputtering system. The magnetron sputtering system is presented in Figure S1. The base pressure in the deposition chamber was 2 × 10^−6^ Torr with Argon (Ar) introduced into the chamber (as a working gas) at a flow rate of 20 sccm. Note that the working pressure was maintained at 3 mTorr and RF power was set at 50 W during pre-sputtering and deposition. Pre-sputtering was a 20 min process to remove contaminants from the target surface. The substrate holder was rotated at 20 rpm throughout the deposition period. In this study, 20–50 nm thickness was chosen as it is thick enough to provide a uniform coverage of coating on the needle. The coverage may not sufficient when the coating is too thin, whereas thicker coating is not preferred because longer deposition time is not cost-effective. We found that this range of thickness can strike a balance between effectiveness and efficiency.

### In vitro cytotoxicity assays using fibroblast cell line model

The cell adhesion characteristics of the TFMG thin films were analyzed in vitro using 3T3 fibroblast cells, which were originally derived from primary mouse embryonic fibroblast cells. Prior to cell culturing, all of the TFMG-coated samples were exposed to UV light (254 nm) for 30 min for sterilization. The 3T3 fibroblast cells were washed using PBS (phosphate buffered saline, UR-PBS001-5 L, Uni Biotech) and detached from the petri dish using trypsin (03-051-5B, 100 mL, Biological industries). A suspension of 3T3 fibroblast cells (density of 1 × 10^5^ cells/well) was then seeded in 12-wells plates and cultured in conditioned DMEM (Dulbecco's Modified Eagle's Medium after being immersed with uncoated or TFMG-coated hypodermic needles.) The fibroblast cells were then cultured and maintained in an incubator at 37 °C under 5% CO_2_ for 24 h.

MTT (Thiazolyl blue tetrazolium bromide, M2128-1g, Sigma-Aldrich) was dissolved in PBS at a concentration of 5 mg/mL. Each culture well was washed once with PBS solution before adding 1 mL of the MTT solution. The cells were then incubated at 37 °C under 5% CO_2_ for 3 h. The resulting formazan crystals in the mitochondria of the cell was then dissolved and extracted using 1 mL DMSO (dimethyl sulfoxide, D2650-100 mL, Sigma-Aldrich) over a period of 10 min. Only the live cells produced purple formazan crystals. Optical density (O.D.) measurements of the extracted DMSO solution were obtained at 570 nm using a spectrophotometer (BMG LABTECH, SPECTROstar Nano, Germany).

### In vitro hemocompatibility test

Whole blood drawn from an artery of New Zealand rabbits was immediately stored in vacutainer tubes. The vacutainer tubes were then subjected to centrifugation (160 g, 10 min) at room temperature. The resulting supernatant (i.e., Platelet-Rich Plasma (PRP)) was harvested using a glass pipette. Freshly harvested PRP was mixed with PBS and assayed in terms of light transmittance at 414 nm using a spectrophotometer. After incubation for 6 min, the specimens were added to PRP-PBS solution for coagulation tests, wherein data points of light transmittance were recorded and at intervals of 10 s.

### In vivo animal study

#### Subcutaneous injection

All of the animal experiments were approved by the National Defense Medical Center’s Institutional Animal Care and Use Committee (Certificate no. IACUC-16-280) in accordance with the ethics standards of the responsible committee. The mice were grouped and housed in polypropylene cages (n = 5 per cage). The vivarium was maintained on a 12 h light:12 h dark cycle, at room temperature (22 ± 1 °C) under relative humidity level of 50 ± 5% with food (laboratory rodent diet, labdiet 5001, USA) and water ad libitum.

Seven-week-old mice (C57BL6/J, male) were used in the subcutaneous injection studies. At the beginning of the experiments, the mice were weighted (avg. 23.8 g/mice) and underwent hair removal in the dorsal area. Anesthesia was performed via intraperitoneal (IP) injection with 40–80 μL of Zoletil 50 to reduce pain. The durability and anti-adhesion performance of the needles was evaluated by performing 20 hypodermic injections on each mouse. The ten mice used in this study were divided into two groups: (1) control group: hypodermic injections administered using bare 23 G needles, (2) experiment group: hypodermic injections were administered using TFMG-coated 23 G needles.

### Intravenous injection

Intravenous injection performance was evaluated by inserting bare or TFMG-coated needles into two different ear veins of the same rabbit over a period of 3 days. All of the injection sites were covered with 3 M Tegaderm and secured with 3 M tape. The rabbits were housed in cages during the subsequent observation period. After a predetermined period of time, the rabbits were sacrificed to enable the dissection and harvesting of ear vein tissue for histological biopsy.

### Preparation of biopsy sections for histological analysis

Each tissue sample was fixed in 10% formaldehyde, dehydrated, placed in a tissue cassette, and embedded in paraffin wax. The infiltration of paraffin into the porous tissue helped to maintain the intrinsic morphology of the tissue and solidified the sample to facilitate thin sectioning. The paraffin blocks were cut into 3- to 5-μm sections for histological analysis^22^. Tissue sections were mounted on slides, stained with hematoxylin–eosin or Masson’s trichrome staining, and viewed and photographed under an optical microscope (OM) (DM 2000; LEICA, Wetzlar, Germany). The area percentage of the injection sites puncturing by needles was estimated using ImageJ software (NIH, Bethesda, MD, USA) as follows, which is based on our previously reported protocol^23^:

$$ {\text{quantitative}}\,{\text{results}}\,{\text{of}}\,{\text{wound}}\,{\text{area}} = {\text{A}}_{{\text{p}}} /{\text{A}}_{{\text{t}}} \times {1}00\% , $$

where A_p_ denotes the wound area at the subscutaneous injection site in each biopsy image, and A_t_ is the total area of the optical microscopic image.

### Statistical analysis

The Student *t* test was used to compare the results obtained from the control and experiment groups. Differences were considered statistically significant when *P* < 0.05. Excel (Microsoft, Seattle, WA, USA) software was used for statistical analysis.

## Supplementary information

Supplementary Figures.
