# Supplementary material for: Preclinical studies of non-stick thin film metallic glass-coated syringe needles
Source: Sci Rep. 2020 Nov 20;10:20313. doi: 10.1038/s41598-020-77008-y (PMC7679377; doi:10.1038/s41598-020-77008-y)
Supplement: Supplementary file 1 — Supplementary Figures. [file 41598_2020_77008_MOESM1_ESM.docx]

Supplementary Materials

# *Full Paper*

**Preclinical studies of non-stick thin film metallic glass-coated syringe needles**

**Running title:** metallic glass-coated syringe needles

Meng-Yi Bai,^1,2^*Ya-Chu Chang,^3^ Jinn P. Chu,^3, 4^*

^1^Graduate Institute of Biomedical Engineering, National Taiwan University of Science and Technology, Taipei 10607, Taiwan (MYB: [mybai@mail.ntust.edu.tw](mailto:mybai@mail.ntust.edu.tw)).

^2^Adjunct appointment to the National Defense Medical Center, Taipei 11490, Taiwan.

^3^Department of Materials Science and Engineering, National Taiwan University of Science and Technology, Taipei 10607, Taiwan (YCC: [m10504311@mail.ntust.edu.tw](mailto:m10504311@mail.ntust.edu.tw)).

^4^Global Taiwan Center for Excellence for Thin-Film Metallic Glass, National Taiwan University of Science and Technology, Taipei, Taiwan. (JPC: [jpchu@mail.ntust.edu.tw](mailto:jpchu@mail.ntust.edu.tw))

*Addresses correspondence to Jinn P. Chu, Ph. D., Department of Materials Science and Engineering, National Taiwan University of Science and Technology, No.43, Keelung Rd., Sec.4, Da'an Dist., Taipei City 10607, Taiwan (e-mail: jpchu@mail.ntust.edu.tw) and Meng-Yi Bai, Ph. D., Graduate Institute of Biomedical Engineering, National Taiwan University of Science and Technology, No.43, Keelung Rd., Sec.4, Da'an Dist., Taipei City 10607, Taiwan (e-mail: mybai@mail.ntust.edu.tw)


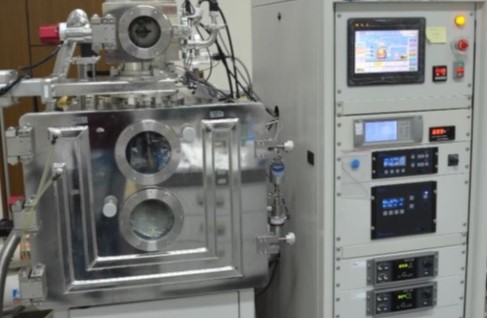


**Figure S1** Photo of a magnetron sputtering system.

| Bare  #26 | front side | 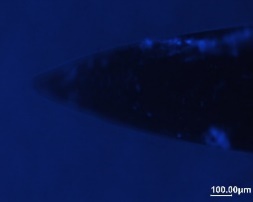 | 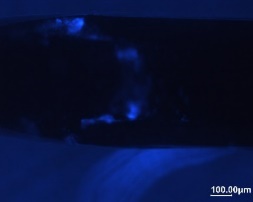 | 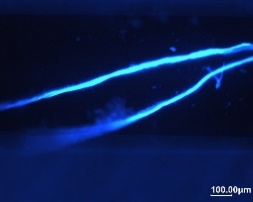 | 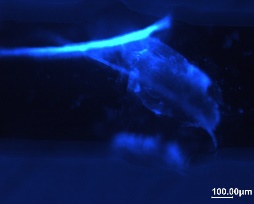 |
| --- | --- | --- | --- | --- | --- |
|  |  | 1.46/22.15 | 1.70/40.84 | 4.93/38.21 | 7.55/38.94 |
|  | back side | 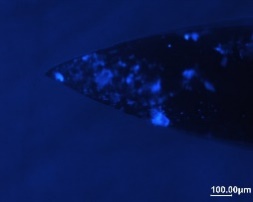 | 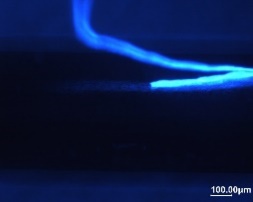 | 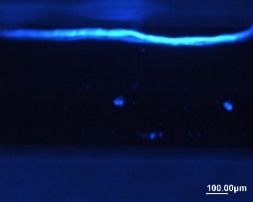 | 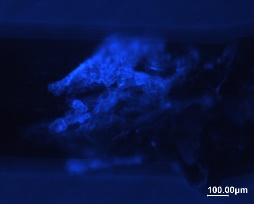 |
|  |  | 1.82/20.73 | 3.24/38.49 | 4.09/39.49 | 7.74/38.74 |
| Bare  #28 | front side | 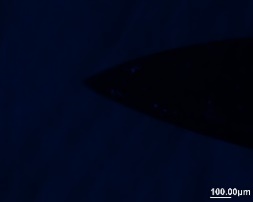 | 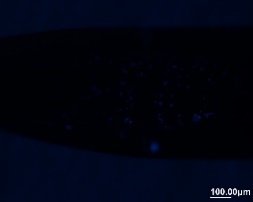 | 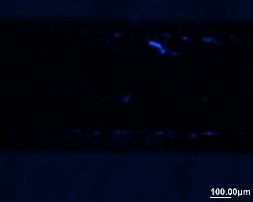 | 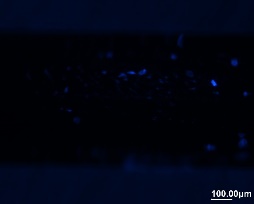 |
|  |  | 0.03/14.93 | 0.57/39.96 | 0.69/41.72 | 1.25/41.56 |
|  | back side | 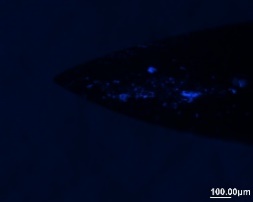 | 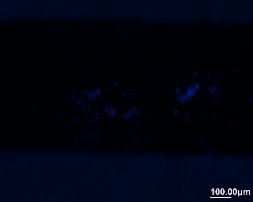 | 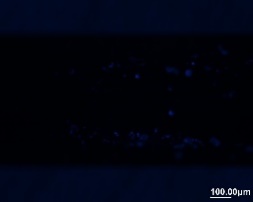 | 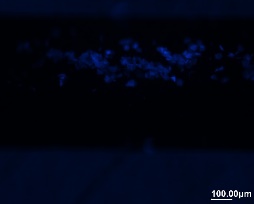 |
|  |  | 0.80/19.78 | 1.18/41.5 | 0.54/43.79 | 2.99/41.23 |
| Bare  #29 | front side | 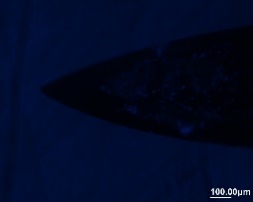 | 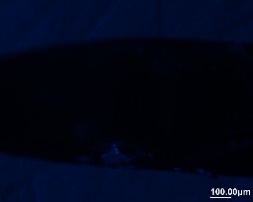 | 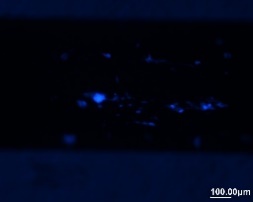 | 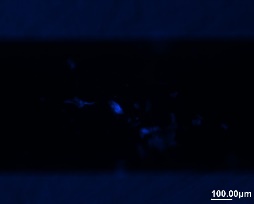 |
|  |  | 2.10/21.16 | 0.52/39.97 | 0.98/41.71 | 1.00/41.6 |
|  | back side | 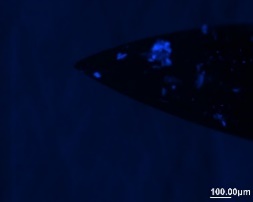 | 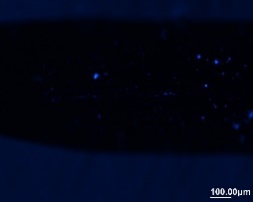 | 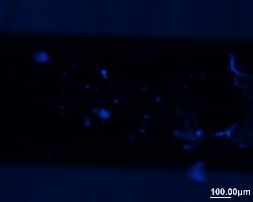 | 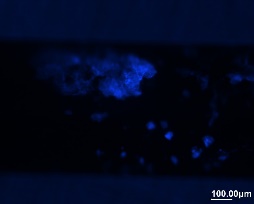 |
|  |  | 1.18/16.98 | 0.80/40.65 | 1.77/42.88 | 4.53/42.31 |

| TFMG  #22 | front  side | 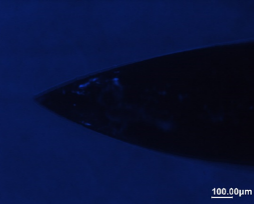 | 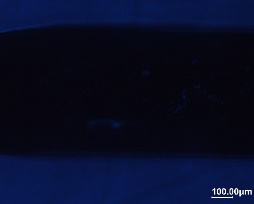 | 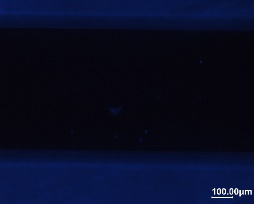 | 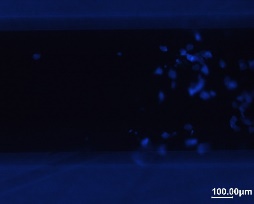 |
| --- | --- | --- | --- | --- | --- |
|  |  | 0.38/22.28 | 0.06/42.87 | 0.27/39.20 | 3.11/39.61 |
|  | back  side | 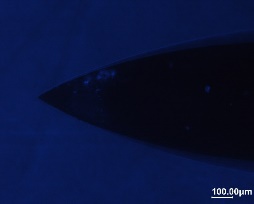 | 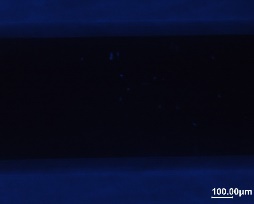 | 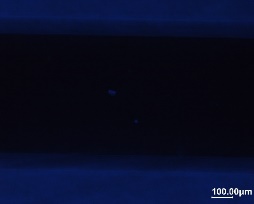 | 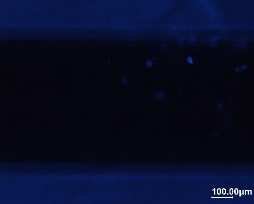 |
|  |  | 0.34/21.31 | 0.47/39.52 | 0.05/38.36 | 0.26/39.76 |
| TFMG #23 | front  side | 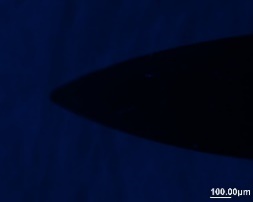 | 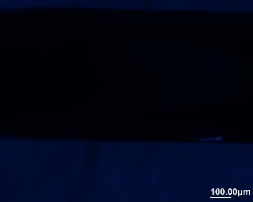 | 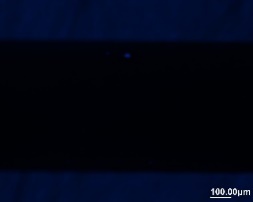 | 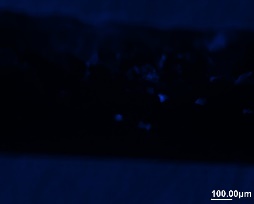 |
|  |  | 0.05/21.37 | 0.11/42.66 | 0.05/42.25 | 1.31/43.84 |
|  | back  side | 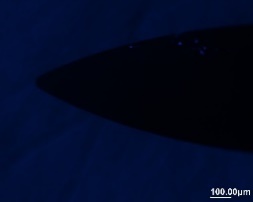 | 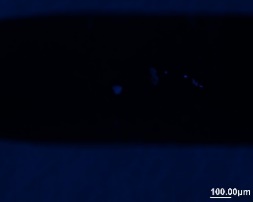 | 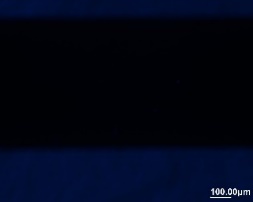 | 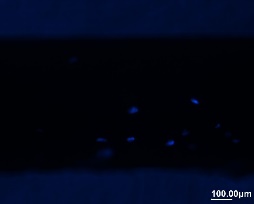 |
|  |  | 0.12/23.41 | 0.16/40.89 | 0.01/42.75 | 0.34/42.88 |
| TFMG  #24 | front  side | 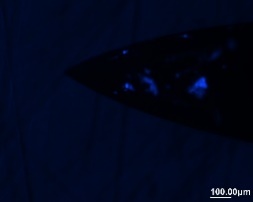 | 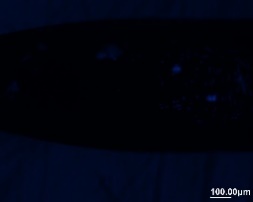 | 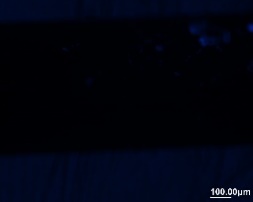 | 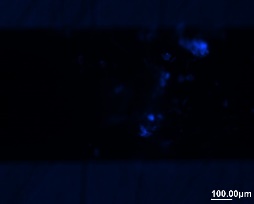 |
|  |  | 0.71/18.2 | 0.64/40.51 | 0.38/42.77 | 1.76/41.75 |
|  | back  side | 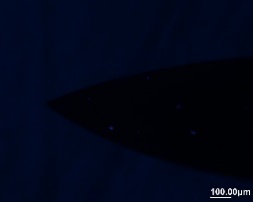 | 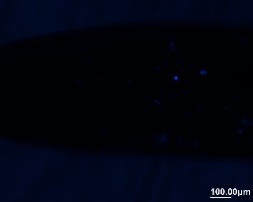 | 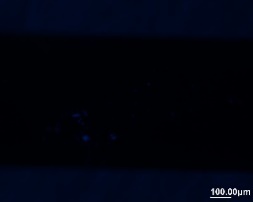 | 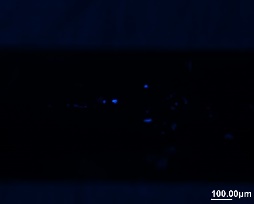 |
|  |  | 0.04/20.45 | 0.24/40.19 | 0.06/44.763 | 0.09/44.836 |

**Figure S2** Raw data used for the statistic results of adhesion cell assessed from the fluorescence area on the DAPI stained needles. Bare and TFMG-coated hypodermic needles were estimated and compared with each other.

| bare |  |  |  |  |
| --- | --- | --- | --- | --- |
|  | 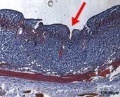 | 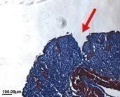 | 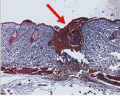 | 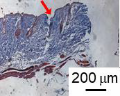 |
|  | 0.16 | 0.82 | 7.08 | 1.13 |
|  | 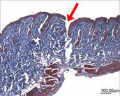 | 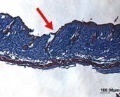 | 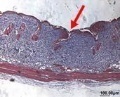 | 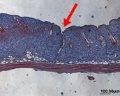 |
|  | 1.35 | 0.28 | 0.90 | 0.52 |
|  | 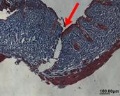 | 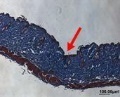 | 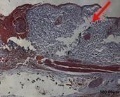 | 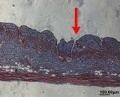 |
|  | 1.88 | 0.17 | 3.09 | 0.13 |
|  | 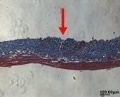 |  |  |  |
|  | 0.07 |  |  |  |
|  |  |  |  |  |

| TFMG |  |  |  |  |
| --- | --- | --- | --- | --- |
|  | 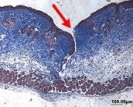 | 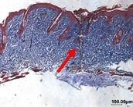 | 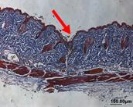 | 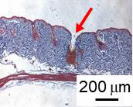 |
|  | 0.67 | 0.61 | 0.26 | 1.80 |
|  | 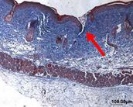 | 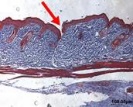 | 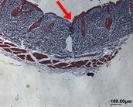 | 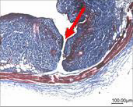 |
|  | 0.28 | 0.28 | 0.53 | 0.95 |
|  | 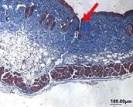 | 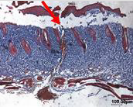 | 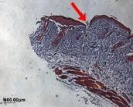 | 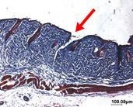 |
|  | 0.68 | 1.42 | 0.13 | 0.56 |
|  | 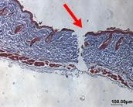 | 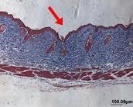 | 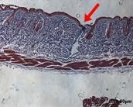 | 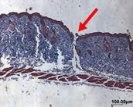 |
|  | 1.45 | 0.41 | 0.49 | 0.99 |

**Figure S3** Raw data utilized for the statistic results of the puncture wound size. The wound size was estimated from all Masson staining images of the mice full skin biopsy shown above.
